# A Rapid FACS-Based Strategy to Isolate Human Gene Knockin and Knockout Clones

**DOI:** 10.1371/journal.pone.0032646

**Published:** 2012-02-29

**Authors:** João F. Mata, Telma Lopes, Rui Gardner, Lars E. T. Jansen

**Affiliations:** Instituto Gulbenkian de Ciência, Oeiras, Portugal; Duke University, United States of America

## Abstract

Gene targeting protocols for mammalian cells remain inefficient and labor intensive. Here we describe FASTarget, a rapid, fluorescent cell sorting based strategy to isolate rare gene targeting events in human somatic cells. A fluorescent protein is used as a means for direct selection of targeted clones obviating the need for selection and outgrowth of drug resistant clones. Importantly, the use of a promoter-less, ATG-less construct greatly facilitates the recovery of correctly targeted cells. Using this method we report successful gene targeting in up to 94% of recovered human somatic cell clones. We create functional EYFP-tagged knockin clones in both transformed and non-transformed human somatic cell lines providing a valuable tool for mammalian cell biology. We further demonstrate the use of this technology to create gene knockouts. Using this generally applicable strategy we can recover gene targeted clones within approximately one month from DNA construct delivery to obtaining targeted monoclonal cell lines.

## Introduction

The ability to locally engineer mammalian genomes represents an invaluable tool to advance mouse genetics, human molecular and cellular biology as well as providing potential to treat genetic defects. The most versatile methodology involves the direct modification of endogenous sequences by gene targeting, allowing either for deleting gene function (knockout) or for the alteration of gene function (knockin). Gene targeting is achieved by providing cells with exogenous DNA that harbor sequences homologous to the genomic target locus resulting in the insertion of foreign sequences or deletion of intervening sequences by homologous recombination. Conventional gene targeting experiments in mouse ES cells or human cell cultures typically require the assembly of large targeting constructs, weeks of drug selection and extensive PCR screening to identify rare recombinants. Targeting frequencies using this methodology are on the order of 1–10 per million transfected cells depending on the extent of homology [1], [2]. Recent advances in the use of zinc finger nuclease technology (ZFN) have greatly improved targeting efficiencies. However, construction of specific nucleases remains either labor intensive or expensive [3]–[5]. In addition, nuclease expression can be genotoxic and lead to undesired mutations [6].

Conventional gene targeting efficiencies have also been significantly improved by the introduction of recombinant Adeno-associated virus (rAAV) mediated delivery of targeting constructs. AAV is a single stranded DNA virus that, in recombinant form, can efficiently deliver a targeting construct to nuclei of a broad range of target cells [2], [7], [8]. rAAV targeting vectors typically have small homology arms that can be easily assembled by PCR-based methods [9]. Although rAAV transduction still leads to frequent random integration, targeting frequencies of up to ∼1% of transduced cells can be achieved by this method [7]. Typically, transduced clones are selected based on acquired resistance to drugs, allowing clonal expansion in culture of positive clones. However, both targeted clones as well as frequent random integration events of the drug resistance cassette are selected by this method, requiring extensive screening to identify targeting events. Targeting frequencies can be further improved by specific vector design. For instance promoter-trap or ATG-trap constructs will express the selectable marker only upon integration into an expressed gene [7], [10] which will often favor selection of correctly targeted clones.

Tagging of proteins with fluorescent proteins has revolutionized cell biology, allowing assessment of protein localization and dynamics in living cells [11]. However, in the majority of cases fusion proteins are overexpressed from constitutive promoters, casting doubt on the physiological relevance of the observed phenomena. These problems are in part ameliorated by the use of BAC transgenesis that mimics endogenous expression although still providing the gene of interest in an ectopic copy and in a foreign chromatin context [12], [13]. We developed a protocol that will allow rapid generation and isolation of human cell clones expressing fluorescent fusion proteins from endogenous loci with minimal perturbations of endogenous gene function. Key to this method is to employ the fluorescence produced by successful targeting as a means of selection of those clones which we term FASTarget (Fluoresence Activated Sorting of Targeted clones). We apply this method to tag or delete the human centromeric histone CENP-A (centromeric protein A) in both transformed as well as non-transformed chromosomally stable human somatic cell lines.

## Results

### Rationale and experimental design

**Figure 1:**
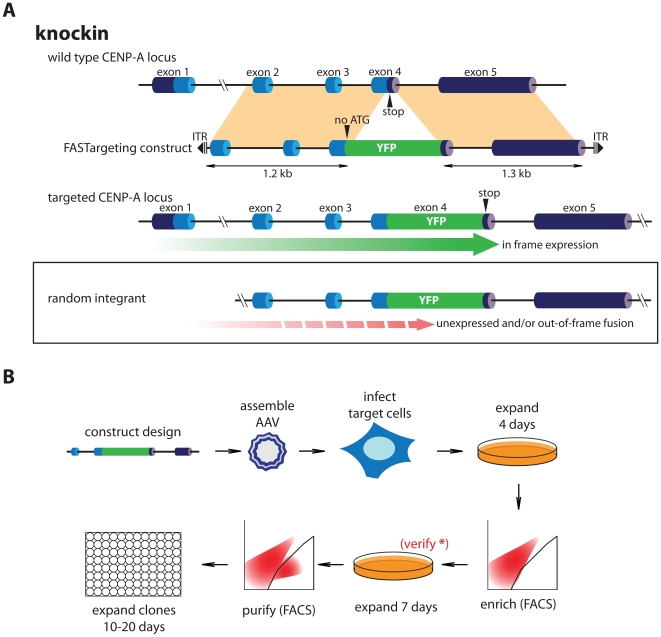
FASTarget rational and experimental outline. (A) Targeting strategy using ATG-less (ORF-trap) EYFP cassette. Genomic structure of the CENP-A locus is outlined. Coding exons in light blue, UTRs in dark blue. Targeting construct is placed within rAAV inverted terminal repeats (ITR) required for transduction. Regions of homology are indicated in beige. Upon targeting the ATG-less EYFP is placed in frame with the last codon of the CENP-A gene resulting in a CENP-A-EYFP knockin driving EYFP fluorescence from the endogenous CENP-A promoter. Random integration will likely result in a failure to express EYFP due to the lack of a nearby promoter or out of frame fusion. (B) Schematic and time-line for rAAV production, transduction, enrichment and purification of targeted clones. Following initial enrichment and expansion cells can be screened as a polyclonal pool by PCR or microscopy to verify (*) presence of targeted cells.

Existing strategies for gene targeting in human cells often require drug selection and clonal outgrowth of selected clones. Typically, drug resistance does not discriminate between targeting events and random integration. We set out to develop an approach to avoid these drawbacks. The method is based on the use of rAAV vectors [2], [14] that feature yellow fluorescent protein (EYFP) as the only foreign DNA to be introduced into target cells flanked by short ∼1 kb homology arms. EYFP is included as a promoter-less and ATG-less (ORF-trap) construct which will result in expression only upon in-frame integration within an expressed gene. This requirement is expected to select against random integration events and will strongly favor correct gene targeting events (Figures 1A and S1). Importantly, EYFP is used as a means for direct selection of targeted clones obviating the need for time consuming outgrowth of drug resistant clones. Cells are selected within days of infection by two rounds of high stringency fluorescence activated cell sorting (FACS) followed by direct inspection of individually sorted clones by microscopy (Figure 1B). This latter step avoids the need for PCR-based methods as a first means of screening for targeting events.

We designed a compact targeting construct consisting of a ATG-less EYFP-tag (carrying a 6×His-2×Prescission tag upstream of EYFP that functions as a linker as well as a protease separable affinity tag [15]) flanked by homology arms that, upon correct targeting, fuses EYFP to the last coding exon of the centromeric histone H3 variant CENP-A (see Figure S1 for sequence details). This gene is encoded by a small 5 exon gene on chromosome 2. The resulting knockin extends the last coding exon with EYFP, fully recapitulating the downstream stop codon and 3′UTR sequences (Figures 1A and S1). CENP-A is a protein with a relatively low abundance allowing us to assess the sensitivity of this methodology. Importantly, functional CENP-A protein localizes in a stereotypical manner to centromere foci [16] that can be easily distinguished by fluorescence microscopy providing a means for visual screening of clones.

### FACS-mediated selection of EYFP knockin clones

**Figure 2:**
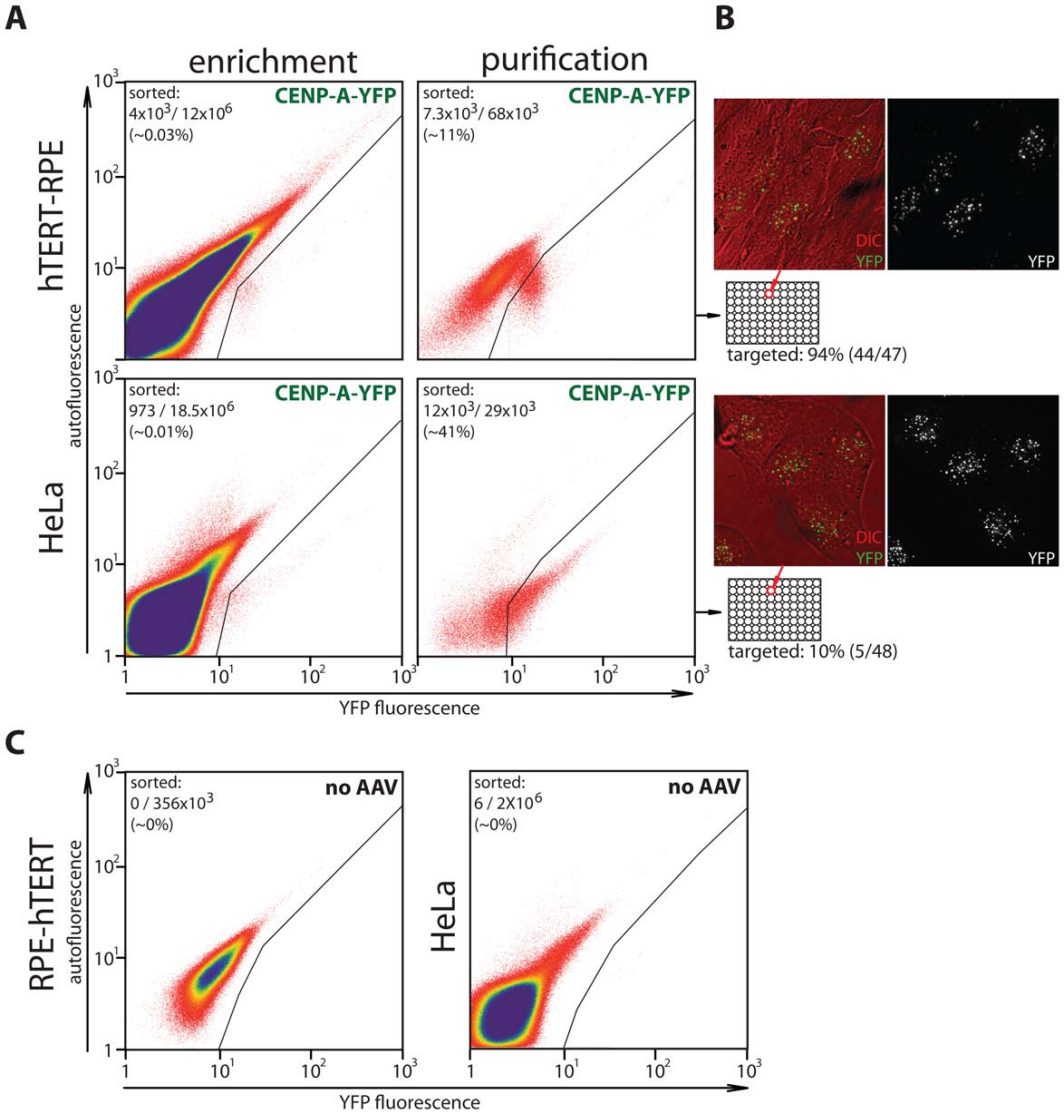
FACS-based strategy to isolate fluorescent knockin clones. (A) Targeting of CENP-A-EYFP knockin construct into human hTERT-RPE or HeLa cells. FACS profiles of the 1^st^ (enrichment) and 2^nd^ (purification) runs are shown. Gates used to collect EYFP positives are shown. Number of cells collected per total number of cells analyzed is indicated for each run. (B) Live cell differential interference contrast (DIC) and epifluorescent (EYFP) image is shown for representative monoclonal isolates for hTERT-RPE (top) and HeLa (bottom). (C) FACS profiles of parental (non-EYFP fluorescent) hTERT-RPE and HeLa cells with gate setting used for purification-mode sorting of rAAV infected cells. Positive hits detected under these conditions represent background fluorescence.

**Figure 3:**
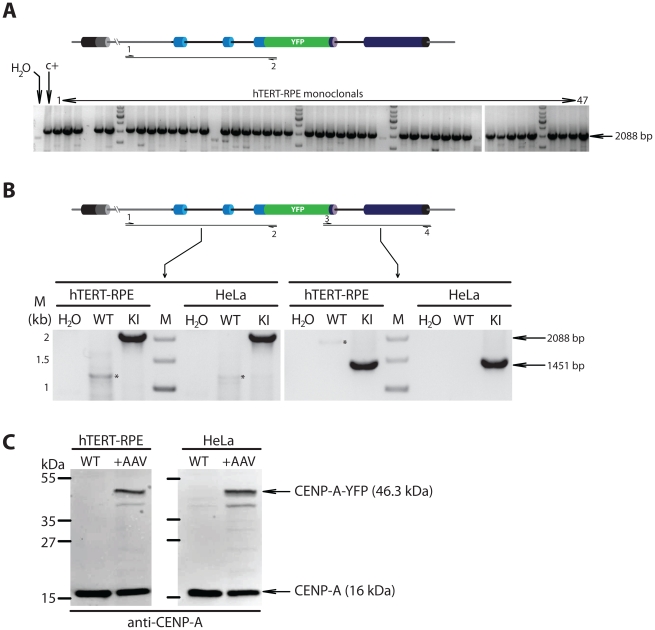
PCR screening and Western verification of EYFP-knockin clones. (A) Schematic outlining PCR screening strategy. Grey regions indicate sequences outside the targeting construct. A primer set in which one primer complementary to a region outside the targeting construct and one primer inside the targeting construct (EYFP) was used to amplify a 2088 bp fragment unique to targeted clones. 47 monoclonal lines produced in the pipeline outlined in Figure 1B were screened for targeting. Water and a previously targeted HeLa clone (c+) were used as negative and positive clones respectively. (B) PCR verification of targeting. Representative clones are shown in which the left and right flanks are amplified using primer set in which one primer anneals outside the targeted area and one to EYFP sequences. Sizes of marker fragments (M) and of expected products are indicated. WT, wild type uninfected parent cells. KI, knockin clones. Asterisks indicate non-specific background products (C) Wild type hTERT-RPE or HeLa or targeted knockin clones were processed for SDS-PAGE and immunoblotting with an anti-CENP-A antibody revealing novel high molecular weight CENP-A-EYFP fusion protein in targeted clones only. Proteins from both the targeted CENP-A-EYFP and untagged CENP-A alleles are detected.

We infected either untransformed hTERT-immortalized retinal pigment epithelial (RPE) cells or HeLa cells, a commonly used cancer cell line, using low-titer rAAV vector particles directly from crude packaging cell lysates (see methods). Following four days of non-selective expansion post infection, cells were sorted for rare acquired EYFP fluorescence (Figure 2A). This sorted population, enriched 3.000–10.000 fold for YPF positive cells, was further expanded for 7 days. At this stage a large proportion of cells expressed EYFP fluorescence (11% and 41% for RPE and HeLa cells respectively). Optionally, at this point the mixed population can be scored for targeting by PCR or by visual inspection (Figure 1B, asterisk). Therefore, potential success can be determined as early as ∼12days after infection. Cells are then re-sorted individually into 96 well plates. Following expansion, monoclonal lines were directly scored for correct EYFP centromere localization by fluorescence microscopy. In this way we found that 94% of recovered RPE clones (44/47) displayed centromeric EYFP fluorescence which was confirmed by PCR genotyping and Western blotting (Figures 2B and 3). Thus, without any prior drug selection we obtained a near homogenous pool of gene targeted cells demonstrating the combined selective power of the vector design and FACS mediated selection of clones. Although targeting in HeLa cells occurred at lower frequencies (10% (5/48)), correctly targeted clones could be readily isolated (Figures 2B, 3B and C).

### Gene disruption by FASTarget

**Figure 4:**
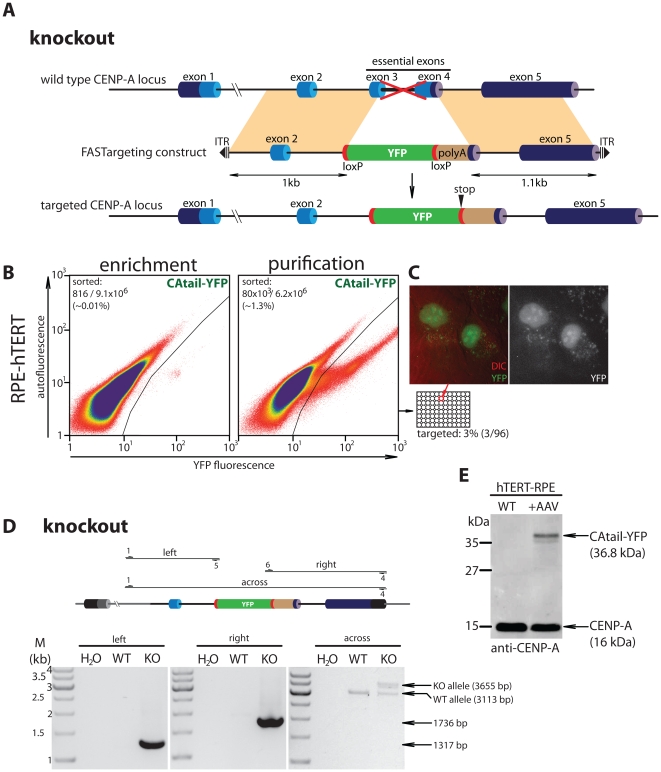
FACS-based strategy to generate a CENP-A knockout allele. (A) Schematic to scale outlining targeting strategy. Correct targeting of the rAAV transduced construct results in the in-frame fusion of EYFP after the first codon of exon 3 and the deletion of the remainder of exon 3 and exon 4. polyA: SV40 poly adenylation signal. (B) Targeting of CENP-A-EYFP knockout (CAtail-EYFP) construct into human hTERT-RPE cells. FACS profiles of the 1^st^ (enrichment) and 2^nd^ (purification) runs are shown. Gates used to collect EYFP positives are indicated. Number of cells collected per total number of cells analyzed is indicated for each run. (C) Live cell differential interference contrast (DIC) and epifluorescent (EYFP) image is shown for a representative monoclonal isolate. Note the loss of centromeric signal and resulting diffuse nuclear EYFP fluorescence. (D) PCR verification of CENP-A knockout. Left and right flanks were analyzed analogous to Figure 3B. Right panel (across): A PCR using two primers flanking the targeted domain resulting in the amplification of both the targeted knockout allele (KO) as well as the untargeted wild type allele (WT). Sizes of marker fragments (M) and of expected products are indicated. All primer numbers correspond to those listed in methods (E) Immunoblot showing targeted CENP-A knockout clone and parental wild type hTERT-RPE cells. Anti-CENP-A antibody reveals a novel high molecular weight species in which a 70 amino acid N-terminal fragment of CENP-A is fused to EYFP (CAtail-EYFP).

Given the applicability of the FASTarget strategy to create knockin clones we reasoned the same approach might facilitate the generation of gene knockouts. Structure-function analysis of CENP-A has shown that a region encompassing loop1 and the alpha-2 helix of the CENP-A histone fold domain are essential for CENP-A function [17], [18]. Removal of this region results in the loss of approximately two-thirds of the entire histone fold resulting in a non-centromere localized non-functional truncated protein. We aimed at deleting the exons encoding this region (3 and 4) and replacing them with the ORF encoding EYFP placed in frame with the endogenous ORF, thereby driving EYFP expression from the endogenous promoter (Figures 4A and S2). In this case, we included loxP sites just upstream and downstream of the EYFP coding sequences allowing optional Cre mediated excision and EYFP recycling for serial targeting. Targeting of this construct results in a premature stop codon that is predicted to trigger non-sense mediated decay of the transcript [19]. To avoid this we introduced an SV40 poly adenylation signal immediately downstream of the novel stop codon. Following infection and two rounds of FACS isolation (Figure 4B), monoclonal hTERT-RPE lines were screened for targeting by PCR. Targeted deletion of CENP-A was detected in 3 out 96 clones (3%). The resulting fluorescent gene product failed to localize to centromeres indicating successful abrogation of CENP-A gene function (Figure 4C). Moreover, correct deletion of targeted exons was confirmed by PCR and the appearance of a truncated CENP-A protein by immunoblot in which the non-functional N-terminal tail is fused to EYFP (Figures 4D and E). Deletion of CENP-A genomic sequences was successful albeit at lower frequencies, consistent with earlier observations that removal of sequences is less favorable than inserting DNA [20].

## Discussion

Fluorescently tagged proteins are widely used in cell biology to determine localization and protein dynamics in living cells [11]. A major caveat is the use of force expression of tagged proteins from ectopic plasmids or from stable integrations of cDNA constructs driven by viral promoters. This raises the concern for potential artifacts affecting protein localization and dynamics. Physiological expression of tagged proteins is essential to properly assess their role in cellular processes. Homologous recombination of exogenous sequences into target genomes allows for the introduction of highly selective genetic changes that include gene knockouts as well as knockin of point mutations or tags. While the efficiency of conventional gene targeting is inherently low we present an approach that allows for rapid and efficient isolation of targeted cell clones.

Selectivity and speed are achieved by combining two specific advances. First, a targeting construct is used that features a promoter-less, ATG-less selection cassette (ORF-trap). In this way, expression depends on in-frame integration of the expression cassette into an active gene. While dependent on target gene expression, this feature selects against random integration events and favors correct gene targeting events. In principle, an ATG-trap construct containing a start codon (but no promoter) would suffice. In that case expression upon random integration will occur only upon integration upstream of the first coding exon of an active gene [2]. Secondly, isolation of selected clones is sped up by employing the fluorescence gained as a result of gene targeting and use it as a means to select cells by FACS within days of targeting construct delivery. This avoids several weeks of drug selection to obtain resistant clones.

Fluorescent reporters as a means for selection have been used before in gene targeting experiments [21], [22]. We now improve on this by employing an ORF-trap in combination with fluorescent proteins to efficiently generate both knockout and functional knockin clones in both transformed (HeLa) and non-transformed genomically stable human RPE cell lines. Importantly, when generating functional knockin clones not only fluorescence but also correct localization is used as a means for selection of targeted clones. The histone H3 variant CENP-A, targeted in this study, localizes in a highly stereotypical manner. This allows for direct microscopy-based screening rather than by PCR to identify targeted clones. This approach is applicable in cases in which a scorable subcellular localization is expected. When this is not the case, traditional PCR screening will be required as, for instance, in the knockout approach described here. Although relatively high frequencies were obtained in targeting CENP-A using a knockin construct (up to 94%), this frequency is likely to vary for different genes depending on DNA sequence and chromatin context. An important parameter is the extent of sequence homology and potential interruptions by SNPs [23]. For instance, in the case of the CENP-A gene, the two alleles in hTERT-RPE differ at 9 positions [single of other simple nucleotide polymorphisms (SNPs)] that are scattered across both homology arms which is predicted to affect targeting efficiency. For this reason, to obtain best results, we sequenced endogenous loci to be targeted and ensure that targeting constructs match the genotype of the target cell line.

We have shown here how gene function can be disrupted by in-frame fusion of YFP to a portion of the protein while deleting essential domains. In principle, homozygous deletions can be obtained by recycling of the YFP selection cassette. Our knockout construct is designed such that YFP, fused in frame with the first two exons of CENP-A is flanked by two loxP sites (Figures 4 and S2) that can be removed by Cre expression. Clones in which YFP is removed can be recovered by FACS. Once YFP-negative clones are isolated and re-grown these can be retargeted bearing in mind possible SNPs between the first and second allele. We expect this procedure to add an additional 2.5 months in order to obtain a double targeted cell line. In summary, we present an improved, rapid FACS-based method that generates functional genomically tagged proteins as well as gene knockouts in human somatic cell lines.

## Materials and Methods

### Gene cloning

A CENP-A-EYFP knockin construct was built by replacing GFP with a PCR generated EYFP (carrying citrine and monomerization mutations: Q69M, A206K) fragment lacking the ATG start codon into the BspEI and XbaI sites of pIC111 [15]. A 5′ homology arm was generated by PCR that covers the CENP-A locus from 43 bp upstream of exon 2 to the last codon of CENP-A in exon 4 spanning a total of 1189 bp. This fragment was cloned as a NheI-SacII containing fragment carrying a NotI site just 3′ from the NheI site. The 3′ homology arm was designed such to reproduce the endogenous CENP-A stop codon and cover the 3′UTR and intronic sequences down to 38 bp upstream of the end of exon 5 spanning a total of 1275 bp. This fragment was cloned as an XbaI fragment carrying a NotI site just 5′ from the end of the right arm. The entire cassette was transferred as a NotI fragment into the corresponding site of pAAV-LacZ [14], replacing the LacZ cassette and inserting the CENP-A-EYFP targeting construct in between the rAAV inverted terminal repeats (ITR) (See Figure S2 for detailed schematic). Targeting results in the formation of a 46.3 kDa fusion of the 6×His-2×Prescission-EYFP tag (LAP-tag [15]) to the last amino acid of CENP-A.

For generation of the CENP-A knockout construct, EYFP (Q69M, A206K) lacking the ATG codon was placed between loxP sites in identical orientation. The 5′ homology arm covers the CENP-A locus from 367 bp upstream of exon 2 and ending just downstream of the 1^st^ codon in exon 3 spanning a total of 996 bp. The 3′ homology arm was placed immediately downstream of the 3′ loxP site which includes the endogenous CENP-A stop codon in exon 4 and reproducing the 3′UTR and part of exon 5 through 1067 bp downstream of the CENP-A stop codon. A PCR generated 251 bp SV40 polyadenylation sequence from pEYFP-N1 (Clontech) was inserted into the unique SacI site just downstream of the CENP-A stop codon (See Figure S2 for detailed schematic). Targeting results in the insertion of a loxP encoded linker fused to EYFP that is in turn fused to the first codon of exon 3 and the deletion of the remaining CENP-A coding sequences. This results in the production of a 36.8 kDa protein in which the first 70 amino acids of CENP-A (CAtail) are fused to EYFP.

### Cell Culture

HeLa cells (gift from Don Cleveland [24]) and their derivatives were cultured in DMEM medium supplemented with 10% heat inactivated newborn calf serum at 37°C 5% CO_2_. hTERT-RPE (gift from Prasad Jallepalli [14]) were cultured in DMEM-F12 medium supplemented with 10% heat inactivated fetal bovine serum and 0.348% Sodium Bicarbonate at 37°C 5%CO_2_. Cells were passaged by a single wash in D-PBS and harvested by trypsinization in 1× TrypLE Express. All Cell culture reagents are from Gibco.

### rAAV production and infection

Targeting constructs are packaged into rAAV particles as described [14]. One day before infection, 1 million RPE-hTERT or HeLa cell lines were seeded in T75 flasks. 48 h after infection excess rAAV particles were removed and cells were expanded for another 2 days before sorting.

### Flow cytometry

rAAV infected or uninfected controls were resuspended in ∼2–4 ml/T75 flask ice cold filter sterilized (0.2 µm) D-PBS supplemented with 5% BSA. Cell aggregates were removed by passage over a 0.45 µm mesh by gravity flow and kept on wet ice at all times.

Cells were sorted on a MoFlo (Beckman Coulter, USA), using a 100 µm nozzle at 206.8 kPa (30 psi) and ∼39 kHz Drop Drive frequency. A 488 nm air-cooled Coherent Sapphire 488-200 CDRH laser (140 mW output) was used for forward and side scatter as well as autofluorescence measurements. EYFP excitation was achieved with a Coherent Innova I90C Argon laser set at 514 nm (220 mW output). Forward scatter was used as trigger and EYFP emission was detected using a 550/30 band-pass emission filter. EYFP (550/30) versus autofluorescence (580/20) signals are plotted defining the autofluorescent ratio. Cells deviating from this correlation in the EYFP channel are scored as EYFP positive. In addition, non-fluorescent cells were used to define the EYFP gate (Figure 2C).

Cells were sorted in “enrich mode” and collected into ∼4 ml of ice cooled collection medium (DMEM/F12 full (Gibco) medium consisting of 50% fresh and 50% conditioned medium (harvested from log phase growth cells and 0.2 µm filtered). Heat inactivated FBS (Gibco) and Fungizone [Amphotericin B (Invitrogen)] were added to a final concentration of 20% and 2.5 µg/ml respectively. Cells were expanded for 7 days and re-sorted as described above except in “single-cell mode” directly into 96-well plates containing 150 µl ice cold collection medium. Gates were set to include false positive hits during the first sorting to enrich for cells with, a priori unknown fluorescent intensities. Gates were set more conservatively during the second sorting run to purify positive clones. Single clones were expanded for 10–20 days.

### Microscopy

To screen for targeting, cells were either transferred to 8 well chambered cover glasses (Lab-Tek) for direct inspection of live cells or onto 12 mm coverslips in 24 well plates, cultured for 24 hours followed by fixation with 4% formaldehyde (Thermo Scientific) for 10 min, permeabilized twice in PBS supplemented with 0.1% triton for 5 minutes and stained with DAPI (Sigma) before mounting in Mowiol (Calbiochem). Digital images were captured using a DeltaVision Core system (Applied Precision) that controls an inverted microscope (Olympus, IX-71), coupled to a Cascade2 EMCCD camera (Photometrics). 512×512 images were collected at 1× binning using a 100× oil objective (NA 1.40, UPlanSApo) with 0.2 µm z sections scanning the entire nucleus. Maximum intensity projections were generated in softWoRx (Applied Precision).

### PCR screening

Cells were harvested by trypsinization and lysed in TNES buffer (10 mM Tris (pH 7.4), 100 mM NaCl, 10 mM EDTA, 0.5% SDS, 100 µg/ml RNase A (Invitrogen)) and incubated for 10 minutes at ambient temperature. DNA was extracted by phenol/chloroform extraction followed by chloroform extraction. Aqueous phase was supplemented with NaOAc to a final concentration of 300 mM and DNA was precipitated with 2 volumes of ethanol, washed in 70% ethanol and resuspended in 10 mM Tris (pH 8), 0.1 mM EDTA.

For PCR a 12.5 µl reaction was assembled containing 16.6 mM Ammonium Sulfate, 67 mM Tris (pH 8.8), 6.7 mM MgCl_2_, 10 mM Beta Mercaptoethanol, 6% DMSO (Sigma), 1.2 mM dNTPs (Fermentas), 10 pmoles of each primer (Sigma), 1 unit of Dream Taq (Fermentas) and ∼200 ng genomic DNA. Reaction was thermo cycled using the following (touchdown) parameters: 94°C, 30 s for 1 cycle; 94°C, 15 s, 63°C for 30 s, 70°C for 2 min (4 cycles); 94°C for 15 s, 60°C for 30 s, 70°C for 2 min (4 cycles); 94°C for 15 s, 57°C for 30 s, 70°C for 2 min (40 cycles); 70° for 6 min; 8°C ∞. Primers used: 1:AGGTGCCTGACACTGACACTGAG, 2:GGGGTAGCGGGCGAAGC, 3:CTGAGCAAAGACCCCAACGAGAAG, 4:CAGTATAATCTCTATCTGTAACAGTAAGTCC, 5:GCTCCTCGCCCTTGCTCAC, 6:AGCAAAGACCCCAACGAGAAGC (see Figures 3A, B and 4D for annotation of primer numbers).

### Immunoblotting

Extracts of ∼10^5^ HeLa or hTERT-RPE cells were separated in an 11% SDS-PAGE gel and transferred to a PVDF membrane. Blots were probed with rabbit anti-human-CENP-A (Cell Signaling #2186) at a dilution of 1∶500. Anti-rabbit HRP-conjugated secondary antibodies were purchased from Jackon Immunoresearch Laboratories.

## Supporting Information

Figure S1 **Nucleotide level schematic of CENP-A-YFP knockin targeting vector.** Inverted terminal repeats (ITR) of the pAAV vector are partially shown. Regions of homology to the CENP-A locus and the YFP containing LAP (6XHIS-PreScission-EYFP) cassette are cloned into NotI sites, internal of the ITRs. Relevant junctions and continuation of reading frame between target locus and the targeting construct are indicated. Nucleotide positions are indicated as a reference for size of the construct.
(TIF)

Figure S2 **As Figure S1 but details for CENP-A knockout (CAtail-YFP) targeting vector are shown.** In this case a recyclable loxP-EYFP-loxP cassette is used and targeted in frame with the first amino acid of exon 3, deleting all downstream coding sequences. PolyA: SV40 polyadenylation signal.
(TIF)
