# Supplementary figures and images for: A Rapid FACS-Based Strategy to Isolate Human Gene Knockin and Knockout Clones
Source: PLoS One. 2012 Feb 29;7(2):e32646. doi: 10.1371/journal.pone.0032646 (PMC3290580; doi:10.1371/journal.pone.0032646)

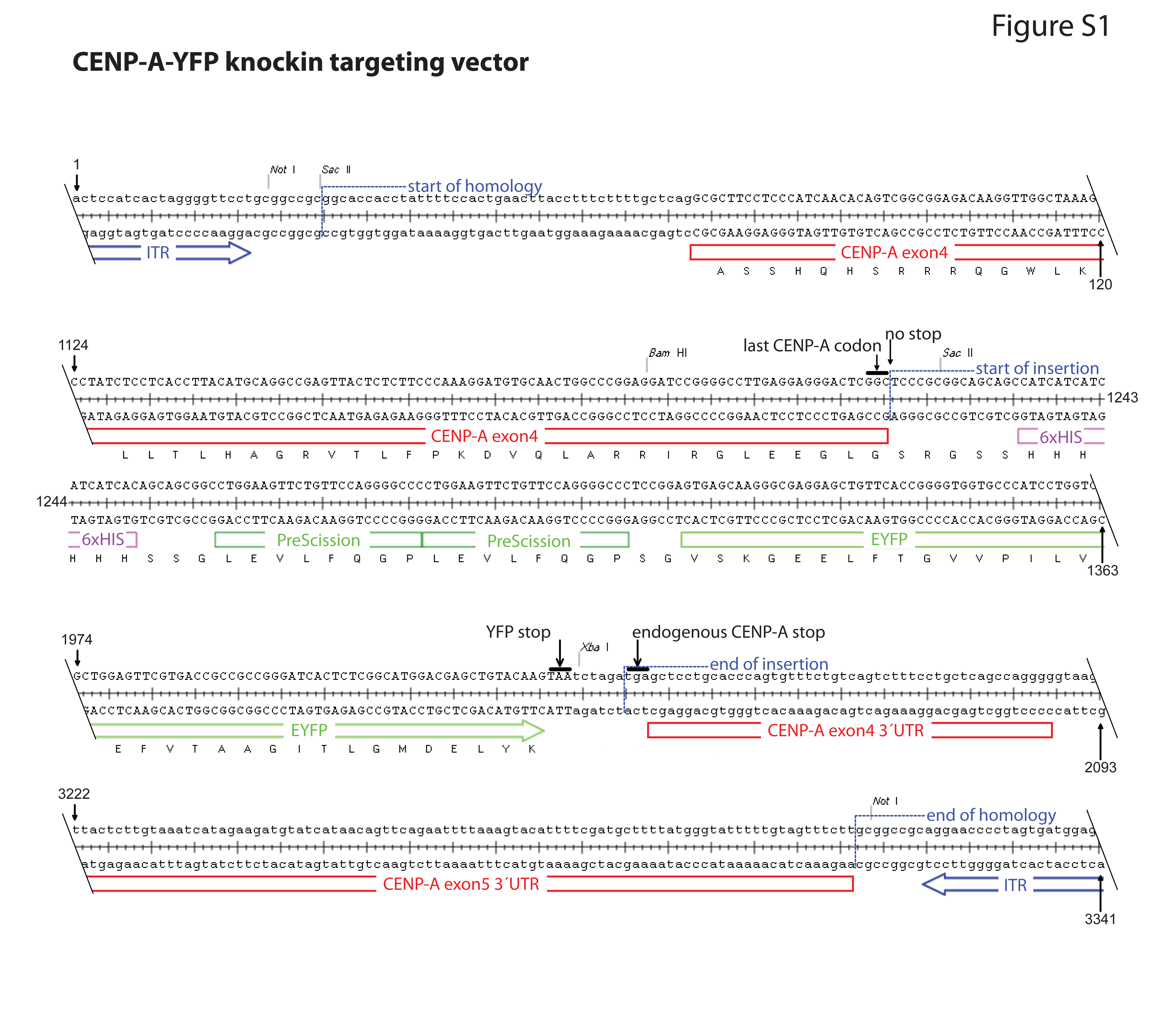

Supplement: Figure S1 — Nucleotide level schematic of CENP-A-YFP knockin targeting vector. Inverted terminal repeats (ITR) of the pAAV vector are partially shown. Regions of homology to the CENP-A locus and the YFP containing LAP (6XHIS-PreScission-EYFP) cassette are cloned into NotI sites, internal of the ITRs. Relevant junctions and continuation of reading frame between target locus and the targeting construct are indicated. Nucleotide positions are indicated as a reference for size of the construct. (TIF) [file pone.0032646.s001.tif]

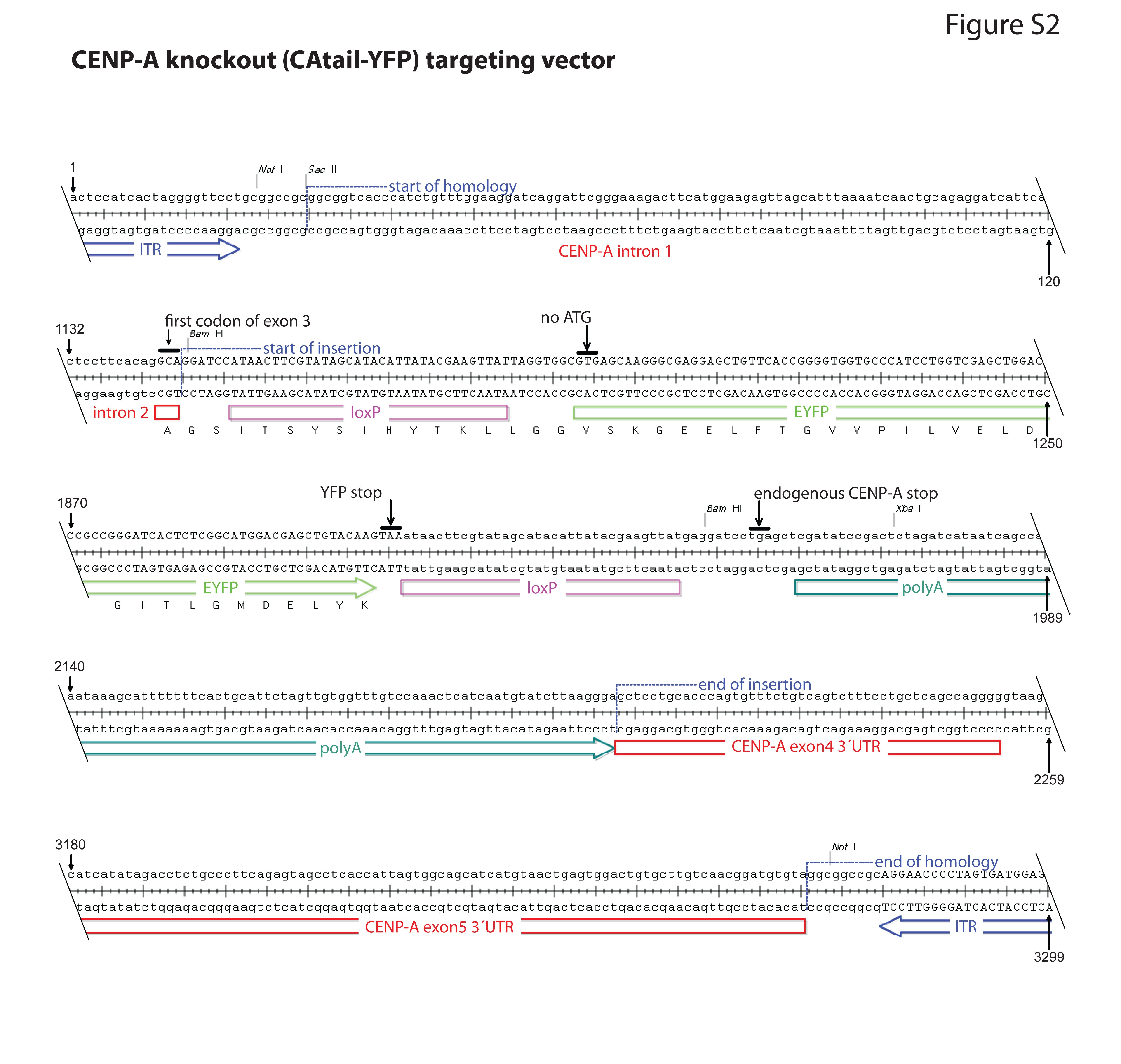

Supplement: Figure S2 — As Figure S1 but details for CENP-A knockout (CAtail-YFP) targeting vector are shown. In this case a recyclable loxP-EYFP-loxP cassette is used and targeted in frame with the first amino acid of exon 3, deleting all downstream coding sequences. PolyA: SV40 polyadenylation signal. (TIF) [file pone.0032646.s002.tif]
